# Prognostic Significance of Time Between Balloon and Peak CK-MB in AMI Patients Undergoing Primary PCI

**DOI:** 10.1016/j.jacasi.2024.12.013

**Published:** 2025-03-11

**Authors:** Eiji Shibahashi, Ryoko Kawakami, Noritoshi Fukushima, Issei Ishida, Hisao Otsuki, Takehiro Hata, Kazuho Kamishima, Kensuke Shimazaki, Takahiro Yamada, Natsuko Shiozaki, Shohei Kataoka, Yuta Morioka, Toshiaki Oka, Yutaka Terajima, Yoshimi Ota, Katsumi Saito, Atsushi Honda, Hiroyuki Tanaka, Junichi Yamaguchi, Kentaro Jujo

**Affiliations:** a Department of Cardiology, Tokyo Women’s Medical University, Tokyo, Japan; b Physical Fitness Research Institute, Meiji Yasuda Life Foundation of Health and Welfare, Tokyo, Japan; c Department of Preventive Medicine and Public Health, Tokyo Medical University, Tokyo, Japan; d Department of Cardiology, Kosei Hospital, Tokyo, Japan; e Department of Cardiology, Nishiarai Heart Center Hospital, Tokyo, Japan; f Department of Cardiology, Tokyo Women’s Medical University Yachiyo Medical Center, Tokyo, Japan; g Department of Cardiology, Sendai Cardiovascular Center, Miyagi, Japan; h Department of Cardiology, Seirei Hamamatsu General Hospital, Shizuoka, Japan; i Department of Cardiology, Saiseikai Kazo Hospital, Saitama, Japan; j Department of Cardiology, Tokyo Metropolitan Tama Medical Center, Tokyo, Japan; k Department of Cardiology, Saitama Medical University, Saitama Medical Center, Saitama, Japan

**Keywords:** acute myocardial infarction, creatine kinase-MB, percutaneous coronary intervention, prognosis

## Abstract

**Background:**

Peak creatine kinase-MB (CK-MB) level is an established predictor of clinical outcomes following acute myocardial infarction (AMI). However, the significance of the duration between balloon inflation and peak CK-MB level (BP time) after primary percutaneous coronary intervention (PCI) remains underexplored in terms of prognostic impact.

**Objectives:**

This study aimed to elucidate the relationship between BP time and prognostic outcomes in patients with AMI.

**Methods:**

In this multicenter observational study, 935 AMI patients who underwent primary PCI and achieved TIMI flow grade 3 on final angiography were included. CK-MB levels were measured systematically at admission and at 3-hour intervals post-PCI. Based on a BP time threshold of 553 minutes, patients were categorized into 2 groups: the long BP-time group (n = 183) and the short BP-time group (n = 752).

**Results:**

The mean age of the patients was 67 years, with a median BP time of 334 minutes (Q1-Q3: 248-491 minutes). The long BP-time group exhibited a higher prevalence of male patients and a history of prior PCI. Cardiovascular mortality was significantly greater in the long BP-time group (log-rank test: *P =* 0.002). Multivariable Cox regression analysis indicated that a prolonged BP time was independently associated with increased cardiovascular mortality (HR: 2.63; 95% CI: 1.19-5.78).

**Conclusions:**

Our findings reveal a significant association between BP time and 1-year cardiovascular mortality in patients with AMI. As a readily assessable parameter, BP time can be a valuable tool for early mortality risk stratification in patients post-primary PCI. (Prognostic Implications of Time between Balloon to Peak Creatinine Kinase-MB in patients with Acute Myocardial Infarction Undergoing Primary PCI: Multicenter Cohort Study; UMIN000049942)

Cardiac biomarkers play a vital role in the clinical diagnosis of acute myocardial infarction (AMI) and in the estimation of infarct size.^1^ Among the widely used biomarkers, the peak values of creatine kinase (CK) and creatine kinase-MB isoenzyme (CK-MB) have been found to be associated with all-cause mortality at 1 year after primary percutaneous coronary intervention (PCI) in patients with AMI.^2^^,^^3^ Therefore, CK-MB values are routinely measured for risk stratification in AMI patients.^4^, ^5^, ^6^ However, the kinetics of released CK-MB are dependent on the status of reperfusion, and it is known that successful reperfusion through PCI leads to higher peak CK-MB values and a quicker time to reach these peak levels.^7^ Consequently, by focusing only on peak CK-MB levels after PCI, there is a potential for confusion regarding whether high peak CK-MB values are a result of poor prognosis or successful reperfusion.

Furthermore, if the time to reach peak CK-MB reflects reperfusion following AMI, it is reasonable to surmise that the time to peak CK-MB may also have an impact on prognosis. However, these relationships have not been adequately validated. To address this gap, this multicenter registry study aimed to investigate the dynamic changes in CK-MB levels after PCI in patients with AMI and examine the prognostic impact of the time from reperfusion by PCI to peak CK-MB. The study is expected to provide insights into the optimal use of CK-MB levels for risk stratification in AMI patients undergoing PCI and improve the prognostic assessment of cardiovascular events in these patients.

## Methods

### Study population, definitions, procedural steps, and endpoints

The GETBACK-AMI (General Estimation of Time between BAlloon and peak CK-MB in prognosis of patients with AMI) cohort study included a total of 1,416 patients with AMI across 8 cardiovascular centers in Japan between April 2011 and April 2017. However, patients who did not undergo primary PCI, those whose onset time of AMI was unknown, and those with a plasma CK level that was already at its peak on admission were excluded. Additionally, patients with TIMI flow grade 0, 1, or 2 on final coronary angiography were excluded to avoid confounding effects on the dynamics of CK-MB values after PCI. Ultimately, 935 patients were analyzed in the study. The study was registered with the University Hospital Medical Information Network-Clinical Trials Registry (UMIN000049942).

AMI was defined as a combination of ischemic symptoms and elevated biomarkers of myocardial injury (troponin) occurring within 48 hours before admission, with or without ST-segment elevation.^8^^,^^9^ The time between balloon and peak CK-MB (BP time) was defined as the time from reperfusion by PCI to peak CK-MB value in patients with AMI. Symptom onset was defined as the onset time, and hospital arrival was defined as the door time.^10^ The time intervals for each event were defined as shown in Central Illustration. PCI procedures were performed according to standard techniques.^9^ Aspirin 100 mg (200 mg loading if no previous medication) and P2Y_12_ inhibitors (clopidogrel: 75 mg daily, 300 mg loading dose if no previous medication; or prasugrel: 3.75 mg daily, 20 mg loading dose if no previous medication) were administered before PCI. The heparin dose during PCI and the use of heparin after PCI were left to the attending physicians at each center. Thrombus aspiration of the culprit lesion was performed when deemed necessary by the operator.

**Central Illustration:**
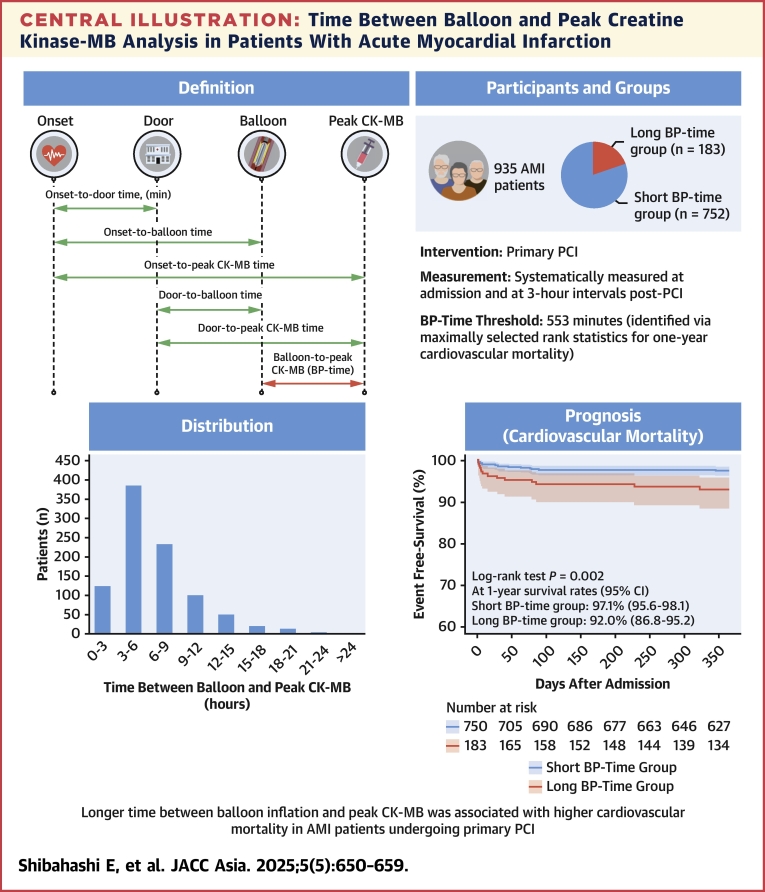
Time Between Balloon and Peak Creatine Kinase-MB Analysis in Patients With Acute Myocardial Infarction The top left panel defines critical time intervals in acute myocardial infarction (AMI) management. The top right panel summarizes the study methodology. The bottom left panel shows the distribution of time between balloon and peak CK-MB (BP time) in the study population. The Kaplan-Meier curves (bottom right) demonstrate that the long BP-time group (red line) had a significantly lower cardiovascular mortality-free survival rate compared with the short BP-time group (blue line). CK-MB = creatine kinase-MB isoenzyme; PCI = percutaneous coronary intervention.

**Figure 1:**
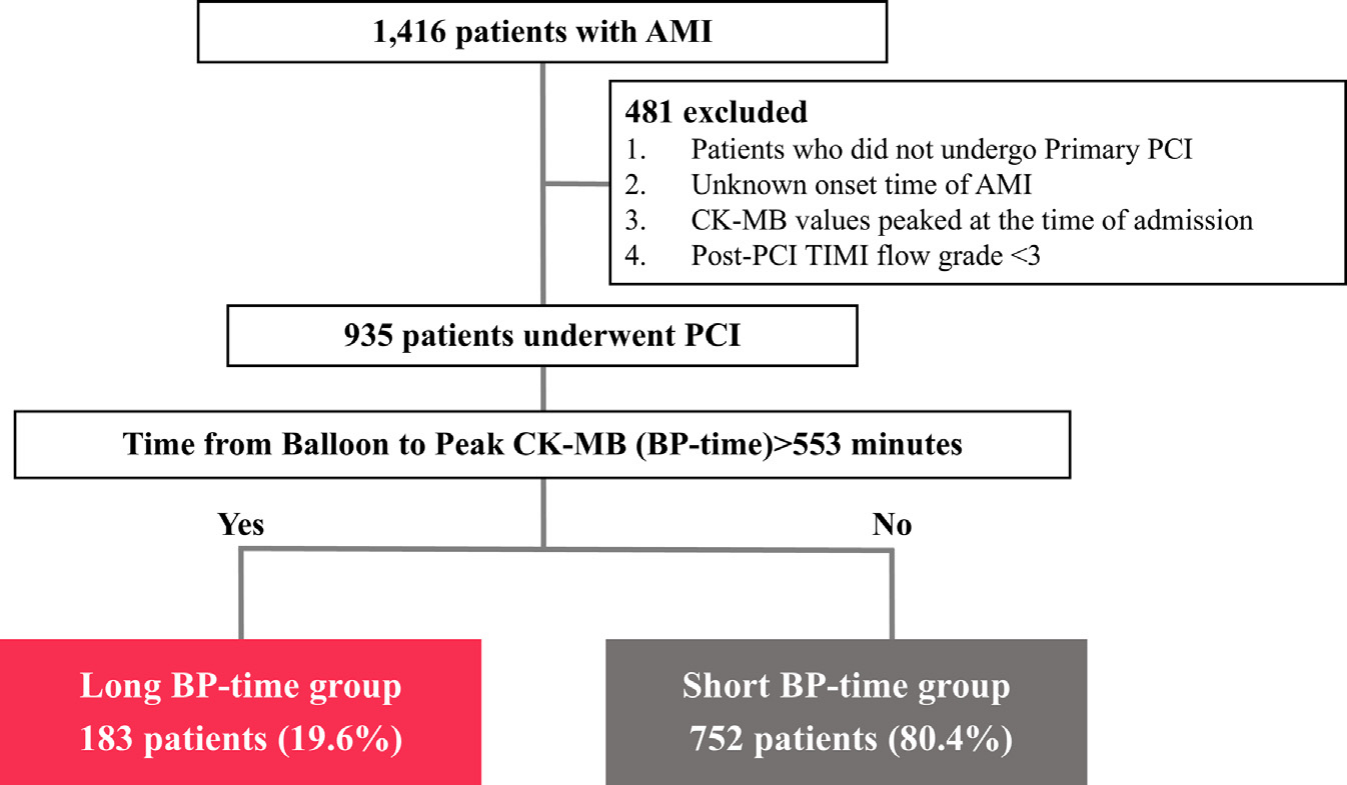
Study Population Of 1,416 acute myocardial infarction (AMI) patients, 935 were included after excluding those without primary percutaneous coronary intervention (PCI), unknown AMI onset time, patients with creatine kinase-MB isoenzyme (CK-MB) values that peaked at the time of admission, or post-percutaneous coronary intervention (PCI) TIMI flow grade <3. Patients were divided into long (n = 183) and short (n = 752) time between balloon and peak CK-MB (BP-time) groups using a 553-minute cutoff.

Serum CK-MB levels were routinely measured on admission and every 3 hours after PCI until confirming the peak value was reached, which was considered the maximum CK-MB value. The primary endpoint of the study was cardiovascular mortality at 1 year. The optimal BP time cutoff point associated with survival outcomes was determined using the maximally selected rank statistics method.^11^ Participants were then divided into long and short BP-time groups based on this cutoff (Figure 1). The long and short BP-time groups were compared in terms of the primary endpoint. The study was carried out according to the principles of the Declaration of Helsinki. The study protocol was approved by the Ethics Committee of Tokyo Women's Medical University (approval number: 4539) on October 26, 2017.

### Statistical analysis

Data are expressed as the mean ± SD and numbers (percentages). The independent Student’s *t*-test and the nonparametric equivalent Mann-Whitney *U* test were used to compare the 2 groups with respect to the continuous variables. The Fisher exact test was used to evaluate categorical variables. Consecutive samples from long and short BP-time patients were compared using the Holm-Sidak method for multiple *t*-tests. To evaluate the association between increased BP time and cardiovascular mortality at 1 year, we conducted a Cox hazard regression analysis. As previously reported, the multivariate model was adjusted for potential confounding factors that might interact with cardiovascular mortality to identify risk factors in patients with AMI, including age, sex, body mass index, systolic blood pressure on admission, plasma hemoglobin value at admission, estimated glomerular filtration rate (eGFR), Killip classification on admission, left ventricular ejection fraction on admission, brain natriuretic peptide, onset-to-door time, door-to-balloon time, and peak CK-MB value.^3^^,^^10^^,^^12^, ^13^, ^14^, ^15^ Missing covariate data were imputed using multiple imputation by chained equations with 20 complete data sets. The patterns of missing data for each variable are detailed in Supplemental Table 1. Moreover, to assess the risk factors correlated with long BP time, univariate logistic regression analyses were conducted on baseline characteristics. Variables achieving a significance level of *P <* 0.10, as well as factors hypothesized to influence BP time, were integrated into the multivariable model. Two-tailed *P* values <0.05 were considered statistically significant. The statistical analyses were performed using R software version 4.4.1 (R Foundation for Statistical Computing).

## Results

Using maximally selected rank statistics, a BP time of 553 minutes was identified as the optimal cutoff for differentiating the groups (Supplemental Figure 1). The study included 935 patients, of whom 183 (19.6%) were classified into the long BP-time group and 753 (80.4%) into the short BP-time group (Figure 1). The median BP time was 334 minutes (Q1-Q3: 248-492 minutes).

### Patient profiles

Baseline patient profiles are listed in Table 1. The average age of this population was 67.0 years, and 76.0% were men. When comparing the long and short BP-time groups, the proportion of male patients, history of PCI, Killip classification, and previous statin use were significantly higher in the long BP-time group than in the short BP-time group (male: 82.5% vs 74.5%; *P =* 0.026; history of PCI: 16.4% vs 10.5%; *P =* 0.029; Killip classification: 1.4 ± 1.0 vs 1.3 ± 0.7; *P =* 0.013; previous statin use: 31.9% vs 23.4%; *P =* 0.022). On the other hand, patients in the long BP-time group had a lower baseline systolic blood pressure, eGFR, and peak levels of CK and CK-MB, and less previous use of thienopyridine compared with those in the short BP-time group (systolic blood pressure: 131.8 ± 34.3 mm Hg vs 137.5 ± 30.7 mm Hg; *P =* 0.041; eGFR: 51.9 ± 25.4 mL/min/1.73 m^2^ vs 59.1 ± 27.2 mL/min/1.73 m^2^; *P =* 0.001; Peak CK: median 1,376 U/L [Q1-Q3: 758-2,598 U/L] vs median 2,315 U/L [Q1-Q3: 1,192-4,270 U/L]; *P* < 0.001; Peak CK-MB: median 133 U/L [Q1-Q3: 69-241 U/L] vs median 221 U/L [Q1-Q3: 120-409 U/L]; *P* < 0.001; thienopyridine: 11.5% vs 18.0%; *P =* 0.036). Additionally, the long BP-time group had a higher TIMI flow grade before PCI, but there was no significant difference in lesion sites treated with PCI between the 2 groups (pre-TIMI flow grade: 1.01 ± 1.14 vs 0.66 ± 0.97; *P* < 0.001) (Table 2).

**Table 1:** Baseline Clinical Profiles

	Long BP-Time Group (n = 183)	Short BP-Time Group (n = 752)	*P* Value
Age, y	66.8 ± 12.0	67.1 ± 12.8	0.781
Male	151/183 (82.5)	560/752 (74.5)	0.026
Body mass index, kg/m^2^	24.0 ± 3.9	23.8 ± 3.9	0.640
Smoking	108/178 (60.7)	422/737 (57.3)	0.447
Hypertension	118/183 (64.5)	497/752 (66.1)	0.728
Dyslipidemia	123/183 (67.2)	458/752 (60.9)	0.126
Diabetes	56/183 (30.6)	272/752 (36.2)	0.168
Atrial fibrillation	9/183 (4.9)	42/752 (5.6)	0.857
Prior myocardial infarction	19/183 (10.4)	50/752 (6.6)	0.113
Prior PCI	30/183 (16.4)	79/752 (10.5)	0.029
Prior CABG	4/183 (2.2)	6/752 (0.8)	0.112
Prior cerebral infarction	12/183 (6.6)	42/752 (5.6)	0.598
Clinical presentation			
Systolic blood pressure, mm Hg	131.8 ± 34.3	137.5 ± 30.7	0.041
Diastolic blood pressure, mm Hg	79.0 ± 22.3	79.0 ± 20.0	0.992
Killip classification	1.4 ± 1.0	1.3 ± 0.7	0.013
1	146/183 (79.8)	657/751 (87.5)	
2	11/183 (6.0)	41/751 (5.5)	
3	9/183 (4.9)	13/751 (1.7)	
4	17/183 (9.3)	40/751 (5.3)	
LVEF, %	53.6 ± 12.4	52.1 ± 11.5	0.166
Laboratory data			
eGFR, mL/min/1.73 m^2^	51.9 ± 25.4	59.1 ± 27.2	0.001
Peak CK, U/L	1,376 (758-2,598)	2,315 (1,192-4,270)	<0.001
Peak CK-MB, U/L	133 (69-241)	221 (120-409)	<0.001
LDL cholesterol, mg/dL	123.7 ± 39.3	123.7 ± 38.9	0.992
HDL cholesterol, mg/dL	48.7 ± 16.0	49.0 ± 19.3	0.851
HbA1c, %	6.4 ± 1.5	6.3 ± 1.3	0.834
Hemoglobin, g/dL	14.0 ± 2.1	14.1 ± 2.1	0.615
BNP, pg/mL	203.8 ± 455.5	164.8 ± 338.2	0.210
Prehospital medications			
ACEI/ARB	54/183 (29.5)	220/752 (29.3)	>0.99
Beta-blocker	26/183 (14.2)	95/752 (12.6)	0.542
MRA	4/183 (2.2)	11/752 (1.5)	0.510
Diuretic	8/183 (4.4)	38/752 (5.1)	0.849
Statin	58/183 (31.9)	176/752 (23.4)	0.022
Aspirin	46/183 (25.1)	181/752 (24.1)	0.773
Thienopyridine	21/183 (11.5)	135/752 (18.0)	0.036
OAC	1/183 (0.5)	23/752 (3.1)	0.065

Values are mean ± SD, n/N (%), or median (Q1-Q3).

ACEI = angiotensin converting enzyme inhibitor; ARB = angiotensin receptor blocker; BNP = brain natriuretic peptide; BP time = time between balloon and peak CK-MB; CABG = coronary artery bypass grafting; CK = creatine kinase; CK-MB = creatine kinase-MB isoenzyme; eGFR = estimated glomerular filtration rate; HbA1c = glycated hemoglobin; HDL = high-density lipoprotein; LDL = low-density lipoprotein; LVEF = left ventricular ejection fraction; MRA = mineralocorticoid receptor antagonist; OAC = oral anticoagulant; PCI = percutaneous coronary intervention.

**Table 2:** Procedural Characteristics

	Long BP-Time Group	Short BP-Time Group	*P* Value
	(n = 183)	(n = 752)	
PCI lesions	1 (1-1)	1 (1-1)	
Left main coronary artery	4/183 (2.2)	17/752 (2.3)	>0.99
Left anterior descending artery	74/183 (40.4)	365/752 (48.5)	0.057
Left circumflex artery	27/183 (14.8)	126/752 (16.8)	0.578
Right coronary artery	76/183 (41.5)	260/752 (34.6)	0.086
Graft	1/183 (0.5)	3/752 (0.4)	0.582
Pre-TIMI flow grade	1.01 ± 1.14	0.66 ± 0.97	<0.001
0	93/183 (50.8)	494/752 (65.7)	
1	16/183 (8.7)	56/752 (7.4)	
2	50/183 (27.3)	170/752 (22.6)	
3	23/183 (12.6)	33/752 (4.4)	
Final TIMI flow grade 3	183/183 (100)	752/752 (100)	NA
Mechanical circulatory support	15/183 (8.2)	48/752 (6.4)	0.410
Onset-to-door time, min	112 (60-232)	138 (65-373)	0.030
Onset-to-balloon time, min	220 (147-360)	248 (159-485)	0.007
Onset-to-peak CK-MB time, min	990 (866-1252)	600 (478-813)	<0.001
Door-to-balloon time, min	81 (60-115)	86 (63-121)	0.251
Door-to-peak CK-MB time, min	818 (731-991)	400 (317-503)	<0.001
BP time, min	715 (632-867)	299 (233-393)	<0.001

Values are median (Q1-Q3) or n/N (%).

Abbreviations as in Table 1.

The long BP-time group had significantly shorter onset-to-door time and onset-to-balloon time than the short BP-time group for each time period (onset-to-door time: median 112 minutes [Q1-Q3: 60-232 minutes] vs median 138 minutes [Q1-Q3: 65-373 minutes]; *P =* 0.030; onset-to-balloon time: median 220 minutes [Q1-Q3: 147-360 minutes] vs median 248 minutes [Q1-Q3: 159-485 minutes]; *P =* 0.007). However, the long BP-time group had significantly longer onset-to-peak CK-MB time, door-to-peak CK-MB time, and BP time than the short BP-time group (onset-to-peak CK-MB time: median 990 minutes [Q1-Q3: 866-1252 minutes] vs median 600 minutes [Q1-Q3: 478-813 minutes]; *P* < 0.001; door-to-peak CK-MB time: median 818 minutes [Q1-Q3: 731-991 minutes] vs median 400 minutes [Q1-Q3: 317-503 minutes]; *P* < 0.001; BP time: median 715 minutes [Q1-Q3: 632-867 minutes] vs median 299 minutes [Q1-Q3: 233-393 minutes]; *P* < 0.001) (Table 2).

### Clinical endpoints

**Figure 2:**
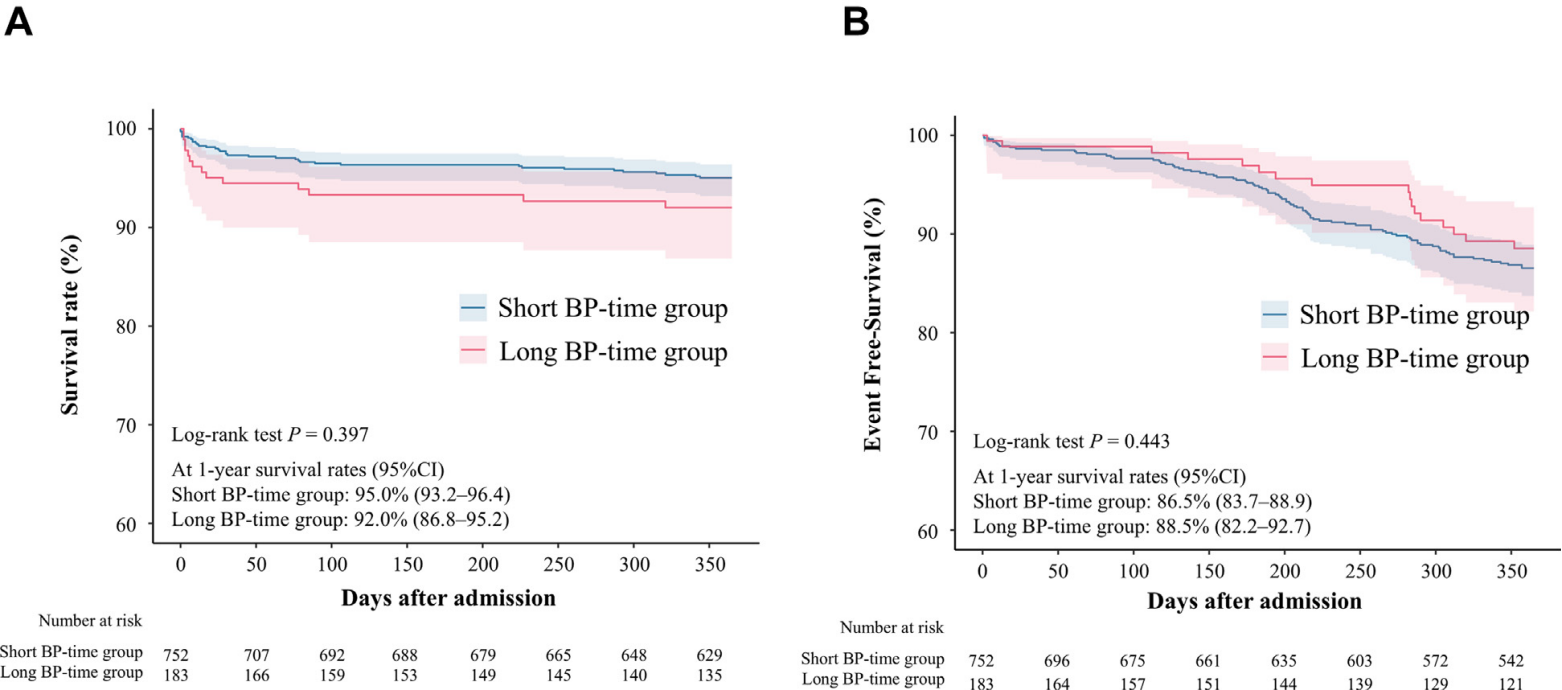
Kaplan-Meier Curves for Clinical Outcomes Kaplan-Meier curves comparing long time between balloon and peak CK-MB (BP-time) (red) and short BP-time (blue) groups for (A) all-cause mortality and (B) target vessel revascularization at 1-year follow-up.

The distribution of BP time revealed that 79.5% of patients had BP time within 9 hours (Central Illustration). Analysis of the BP-time groups showed a distinct pattern: the short BP-time group predominantly consisted of patients whose peak CK-MB values occurred between 3 and 6 hours postballoon inflation, whereas the long BP-time group had a higher frequency of patients reaching peak CK-MB values later, between 9 and 12 hours after the procedure (Supplemental Figure 2). The incidence of cardiovascular mortality was significantly higher in the long BP-time group compared with the short BP-time group (log-rank test: *P =* 0.002), as shown by Kaplan-Meier curves, whereas the incidence of all-cause mortality or target vessel revascularization was not significantly different between the 2 groups (Central Illustration, Figures 2A and 2B). Additionally, the in-hospital mortality rates were comparable in the 2 groups (long BP-time group 7.1% vs short BP-time group 5.7%; *P =* 0.292) (Supplemental Table 2).

Considering the dynamic changes in CK-MB, we conducted an additional analysis using peak CK-MB time as the baseline for the start of follow-up. This analysis showed that the incidence of cardiovascular mortality and its impact on prognosis were consistent with those observed when using admission as the start of the follow-up period (Supplemental Figure 3, Supplemental Table 3).

### Prognostic factors for cardiovascular mortality

In the multivariable Cox analysis assessing prognostic factors for cardiovascular mortality, long BP time emerged as an independent predictor, irrespective of peak CK-MB values. The HR was 2.63 (95% CI: 1.19-5.81) (Table 3).

**Table 3:** HRs of Cardiovascular Mortality at 1-year According to Balloon to Peak CK-MB Time

	Balloon to Peak CK-MB time	
	Short (≤553 min)	Long (>553 min)
Model 1	1.00 (reference)	2.96 (1.50-5.83)
Model 2	1.00 (reference)	2.39 (1.07-5.35)
Model 3	1.00 (reference)	2.63 (1.19-5.81)

Values are HR (95% CI). Model 1: adjusted for age (years) and sex (male, female). Model 2: adjusted for model 1 covariates plus body mass index (kg/m^2^), systolic blood pressure (mm Hg), hemoglobin (g/dL), eGFR (mL/min/1.73 m^2^), LVEF (%), Killip classification (1 to 4), BNP (pg/mL), onset-to-door time (minutes), door-to-balloon time (minutes). Model 3: adjusted for model 2 covariates plus peak CK-MB (U/L).

Abbreviations as in Table 1.

Supplemental Table 4 presents the findings from logistic regression analyses, identifying the predictive factors associated with classification into the long BP time category. Multivariable logistic analysis identified male sex, high Killip classification at admission, the high pre-TIMI flow grade, and non-left anterior descending (LAD) culprit lesion as independent predictors (male: OR: 1.83; 95% CI: 1.02-3.30; Killip classification: OR: 1.30; 95% CI: 1.04-1.62; pre-TIMI flow grade: OR: 1.39; 95% CI: 1.17-1.65; LAD culprit lesion: OR: 0.65; 95% CI: 0.45-0.95) (Supplemental Table 5).

## Discussion

The current study demonstrated that prolonged BP time was associated with increased cardiovascular mortality at 1 year in AMI patients who achieved a final TIMI flow grade 3 post-PCI. This association highlights the potential of BP time as a significant prognostic marker in the context of AMI management. To the best of our knowledge, this is the first study to specifically focus on the relationship between BP time and long-term prognosis in patients undergoing primary PCI for AMI. This finding is particularly important given the current emphasis on rapid revascularization in AMI treatment protocols. Our results suggest that, beyond the achievement of TIMI flow grade 3, the duration between reperfusion and peak cardiac biomarker levels may offer additional prognostic information. This study opens avenues for further research to understand the underlying mechanisms that contribute to prolonged BP time and its impact on patient outcomes. Additionally, it underscores the need to consider BP time in the stratification of risk and tailoring of post-PCI management strategies for AMI patients.

### Relationship between CK-MB and prognosis in AMI

The correlation among peak CK-MB levels, its time–concentration curve area after primary PCI, and 1-year cardiovascular events in AMI patients is well-documented.^2^^,^^3^^,^^16^ Our study contributes to this body of knowledge by highlighting the prognostic significance of the duration from reperfusion to peak CK-MB value. Notably, the findings revealed that patients with longer time from reperfusion to peak CK-MB (long BP-time group) demonstrated poorer 1-year prognoses despite lower peak CK-MB values than their counterparts in the short BP-time group. This indicates a potentially overlooked high-risk AMI subgroup, whose prognosis might be underestimated if assessed solely on peak CK-MB values. Furthermore, the dynamics of CK-MB release are significantly influenced by the state of reperfusion. Previous research has demonstrated that successful reperfusion, as indicated by a favorable TIMI flow grade post-PCI, enhances the washout of CK-MB, resulting in an earlier peak in CK-MB levels.^7^ Previous studies exploring this relationship, however, included approximately 10% of patients who did not achieve TIMI flow grade 3 post-PCI.^2^^,^^3^^,^^16^ This inclusion complicates the ability to precisely determine the relationship between revascularization success and CK-MB values. By excluding patients who did not reach TIMI flow grade 3 in our study, we were able to assess the association more accurately between reperfusion success and CK-MB kinetics. Future research should continue to examine the intricate relationships among peak CK-MB value, the area under its time concentration curve, and the duration from reperfusion to peak CK-MB value in AMI patients undergoing PCI. Such studies will be instrumental in refining our understanding of CK-MB’s role as a prognostic marker in the context of successful revascularization.

### Relationship between time and prognosis

The critical nature of minimizing ischemia duration in AMI, particularly in ST-segment elevation myocardial infarction (STEMI) cases where reperfusion within 90 minutes of diagnosis is recommended, is well-established.^9^ Additionally, the onset-to-door time is known to affect prognosis, highlighting the necessity of reducing this duration.^10^^,^^17^ Despite the emphasis on these time frames, indicators tracking time trends postreperfusion are yet to be established. Our study explored various time intervals (onset-to-door time, onset-to-balloon time, onset-to-peak CK-MB time, door-to-balloon time, door-to-peak CK-MB time) including BP time, as potential markers for post-reperfusion periods. The findings reveal that a prolonged BP time correlates with increased cardiovascular mortality at 1 year, while in our specific cohort, the impact of door-to-balloon time on prognosis was less apparent. The lack of prognostic significance of door-to-balloon time in our study could be attributed to confounding factors such as total ischemic duration, survivor bias, and variations in myocardial necrosis progression, consistent with prior research.^18^, ^19^, ^20^ Additionally, our study's focus on patients achieving TIMI flow grade 3 post-PCI and its relatively small sample size may have contributed to these findings.

Large-scale studies have previously shown that metrics like onset-to-balloon time and door-to-balloon time are crucial for evaluating short-term outcomes, such as in-hospital mortality.^21^^,^^22^ However, our study introduces BP time as a novel index, more relevant for predicting 1-year cardiovascular mortality post-AMI, rather than for stratifying in-hospital mortality risk. This suggests that time-to-reperfusion metrics and postreperfusion peak CK-MB levels (such as BP time) may serve as distinct prognostic indicators. Although minimizing time to reperfusion remains essential for enhancing short-term outcomes in patients with AMI, our findings suggest additional interventions might be necessary to improve long-term outcomes. Our analysis uncovered multiple risk factors contributing to extended BP time, such as male sex, elevated Killip class upon admission, high pre-TIMI flow grade, and non-LAD culprit lesions. Particularly, patients with advanced Killip classification, notably those experiencing congestive heart failure, exhibited a greater likelihood of prolonged BP time. In such cases, close monitoring of the interval to peak CK-MB levels following PCI is recommended to better predict and manage potential complications. Moreover, non-LAD culprit lesions emerged as significant predictors of BP time extension. The presence of non-LAD culprit lesions in AMI should prompt caution rather than a rapid dismissal of risk. Consequently, assessing BP time can be instrumental in identifying high-risk AMI patients with non-LAD culprit lesions, underscoring the importance of this metric in patient risk stratification. The association between favorable pre-TIMI flow grades and prolonged BP time was an unexpected finding, considering that pre-TIMI flow grades vary depending on AMI presentation, whether as STEMI or non-ST-segment elevation myocardial infarction (NSTEMI).^23^ Our study's lack of differentiation between STEMI and NSTEMI might have introduced certain biases. Therefore, further research is warranted to elucidate the relationship between BP time and pre-TIMI flow grades in different AMI presentations.

### Possible mechanisms underlying BP time and prognosis

Initial evaluation of coronary arteries in AMI has traditionally emphasized the assessment and optimization of epicardial blood flow. However, emerging research has challenged this focus, showing that normal epicardial blood flow does not always equate to adequate myocardial tissue perfusion.^24^^,^^25^ In AMI patients who achieve a TIMI flow grade 3 post-PCI, the delayed interval from reperfusion (balloon inflation) to the peak circulating levels of cardiac biomarkers (specifically peak CK-MB) might signify underlying abnormalities in myocardial perfusion. Although this hypothesis remains speculative, it introduces the concept that BP time could potentially reflect the extent of washout phenomena associated with myocardial perfusion. Previous studies have highlighted an association between increased mortality rates and myocardial perfusion abnormalities, particularly microvascular obstruction, in the aftermath of STEMI.^26^ This link was further corroborated in the ACUITY(Acute Catheterization and Urgent Intervention Triage Strategy) trial, where an analysis confined to patients with TIMI flow grade 3 revealed that diminished myocardial perfusion, as denoted by the myocardial blush grade, correlated with elevated mortality rates.^25^ Given these findings, further research is imperative to elucidate the relationship between BP time and indicators of compromised myocardial perfusion. Advanced diagnostic methods, including cardiac magnetic resonance, positron emission tomography, or the index of microcirculatory resistance, could provide valuable insights into this relationship. Such investigations could significantly enhance our understanding of the mechanisms behind BP time as a prognostic marker in AMI, offering potential avenues for more targeted therapeutic strategies.

### Study limitations

Our study contributes to the understanding of BP time in patients with AMI but has several limitations. The biomarker for myocardial injury was measured using CK-MB, but more sensitive indicators of myocardial injury such as troponin T and I were not assessed. Additionally, the accuracy of symptom onset timing, a crucial factor in our analysis, might have been compromised because it relied on the recollections provided by patients and first responders. This subjective nature of reporting could lead to potential inconsistencies and inaccuracies. Moreover, the study encompasses AMI patients as a whole without differentiating between STEMI and NSTEMI. This broad categorization may overlook distinct clinical characteristics and outcomes inherent to each subtype. We also did not have data on procedural times, intraoperative slow flow phenomena, thrombus burden, or the use of adjunctive devices such as aspiration catheters or mechanical thrombectomy. Additionally, information on the prevalence of multivessel disease or valvular dysfunction was unavailable for analysis. Furthermore, we did not assess the myocardial blush grade at the end of the procedure. Evaluation of myocardial blush grade could have provided a more comprehensive understanding of the relationship between BP time and myocardial perfusion status. Survivor bias and variations in post-PCI management among different facilities might also have influenced the results. These limitations, along with unmeasured confounders, could affect the study's conclusions and the extrapolation of its findings. Additionally, the observational nature of the study poses inherent limitations. Differences in baseline characteristics between the long and short BP-time groups might have influenced the observed outcomes, limiting the ability to draw definitive comparisons between these groups.

## Conclusions

This study has highlighted the significance of BP time in the context of AMI. AMI patients in the long BP-time group showed higher incidence of cardiovascular mortality at 1 year compared with those in the short BP-time group. This association establishes prolonged BP time as an independent predictor of cardiovascular mortality. Based on these findings, we advocate for the routine monitoring of CK-MB levels following PCI. Such follow-up could play a crucial role in enhancing risk stratification, potentially leading to more tailored and effective treatment strategies for patients with AMI.

## Funding Support and Author Disclosures

This study was not financially supported by any company, grant, or fund. The authors have reported that they have no relationships relevant to the contents of this paper to disclose.
