# Supplementary material for: Prognostic Significance of Time Between Balloon and Peak CK-MB in AMI Patients Undergoing Primary PCI
Source: JACC Asia. 2025 Mar 11;5(5):650–9. doi: 10.1016/j.jacasi.2024.12.013 (PMC12081220; doi:10.1016/j.jacasi.2024.12.013)
Supplement: Supplemental Figures 1-3 and Supplemental Tables 1-5 [file mmc1.docx]

**Supplemental Figure 1. The standardized log-rank statistics for the BP time**

The analysis identified 553 minutes as the cutoff point, corresponding to the maximum value on the vertical axis (M = 1.6364), dividing participants into groups with BP times ≤553 minutes and >553 minutes.

BP-time = time between balloon and peak CK-MB

**Supplemental Figure 2. BP-time distribution in long BP-time and short BP-time groups**

Bar graph comparing the BP-time distribution between the long BP time group (Red) and the short BP time group (black).

BP-time = time between balloon and peak CK-MB; CK-MB = creatine kinase-myoglobin binding.

**Supplemental Figure 3** **Kaplan-Meier curves for cardiovascular mortality with follow-up baseline at peak CK-MB time**

BP-time = time between balloon and peak CK-MB; CI = confidence interval

**Supplemental Table 1. Missing data patterns**

| **Variable** | **n** | **N (%) cases with missing values** |
| --- | --- | --- |
| Age | 935 | 0 (0) |
| Sex | 935 | 0 (0) |
| Body mass index | 890 | 45 (4.8) |
| Systolic Blood pressure | 833 | 102 (10.9) |
| Diastolic Blood pressure | 831 | 104 (11.1) |
| Killip classification | 934 | 1 (0.1) |
| LVEF | 825 | 110 (11.8) |
| eGFR | 935 | 0 (0) |
| Peak CK-MB levels | 935 | 0 (0) |
| Hemoglobin | 935 | 0 (0) |
| BNP | 864 | 71 (7.6) |
| Onset-to-door time | 934 | 1 (0.1) |
| Onset-to-balloon time | 934 | 1 (0.1) |
| Onset-to-peak CK-MB time | 934 | 1 (0.1) |
| Door-to-balloon time | 934 | 1 (0.1) |
| Door-to-peak CK-MB time | 934 | 1 (0.1) |
| Balloon-to-peak CK-MB time | 935 | 0 (0) |
| All-cause mortality within a year | 935 | 0 (0) |
| Cardiovascular mortality within a year | 935 | 0 (0) |

BNP = brain natriuretic peptide; eGFR = estimated glomerular filtration rate; LVEF = left ventricle ejection fraction; CK-MB = creatine kinase-myoglobin binding.

**Supplemental Table 2. Clinical outcomes in long BP-time and short BP-time groups**

|  | **Long BP-time group**  **n= 183** | **Short BP-time group**  **n = 752** | ***P* value** |
| --- | --- | --- | --- |
| Cardiovascular mortality, % | 14 (7.7) | 21 (2.8) | 0.002 |
| All-cause mortality, % | 14 (7.7) | 36 (4.8) | 0.397 |
| TVR, % | 17 (9.3) | 91 (12.1) | 0.443 |
| In hospital mortality, % | 13 (7.1) | 43 (5.7) | 0.292 |

BP-time *=* time between balloon and peak CK-MB; TVR = target vessel revascularization

**Supplemental Table 3. Hazard ratios for cardiovascular mortality at 1 year according to BP-time levels, with baseline set at Peak CK-MB.**

|  | **Balloon to Peak CK-MB time** | |
| --- | --- | --- |
|  | **Short (≤553 minutes)** | **Long (>553 minutes)** |
|  | HR (95%CI) | HR (95%CI) |
| Model 1 | 1.00 (reference) | 2.96 (1.50-5.83) |
| Model 2 | 1.00 (reference) | 2.38 (1.06-5.32) |
| Model 3 | 1.00 (reference) | 2.62 (1.19-5.78) |

Model 1: adjusted for age (years) and sex (male, female).

Model 2: adjusted for model 1 covariates plus body mass index (kg/m^2^), systolic blood pressure (mmHg), hemoglobin (g/dL), eGFR (mL/min/1.73m^2^), LVEF (%), Killip classification (1 to 4), BNP (pg/mL), onset-to-door time (minutes), door-to-balloon time (minutes).

Model 3: adjusted for model 2 covariates plus peak CK-MB (U/L).

BNP = brain natriuretic peptide; CI = confidence interval; CK-MB = creatine kinase-myoglobin binding; eGFR = estimated glomerular filtration rate; HR = hazard ratio; LVEF = left ventricle ejection fraction.

**Supplemental Table 4. Univariate logistic regression model for long BP-time**

|  | **OR** | **95%CI** | ***P* value** |
| --- | --- | --- | --- |
| Age, years | 1.00 | 0.99-1.01 | 0.781 |
| Male | 1.62 | 1.07-2.45 | 0.023 |
| Body mass index | 1.01 | 097-1.05 | 0.640 |
| History of smoking | 1.15 | 0.82-1.61 | 0.408 |
| Hypertension | 0.93 | 0.66-1.31 | 0.681 |
| Dyslipidemia | 1.32 | 0.94-1.85 | 0.115 |
| Diabetes mellitus | 0.78 | 0.55-1.10 | 0.157 |
| Atrial fibrillation | 0.87 | 0.42-1.83 | 0.722 |
| Hemodialysis | 1.39 | 0.64-3.01 | 0.404 |
| Prior myocardial infarction | 1.63 | 0.93-2.83 | 0.086 |
| Prior PCI | 1.67 | 1.06-2.63 | 0.027 |
| Clinical presentation |  |  |  |
| Systolic blood pressure, mmHg | 0.99 | 0.99-1.00 | 0.042 |
| Diastolic blood pressure, mmHg | 1.00 | 0.99-1.01 | 0.992 |
| Heart rate, beats/min | 1.00 | 0.99-1.01 | 0.981 |
| Killip classification | 1.30 | 1.09-1.56 | 0.004 |
| LVEF, % | 1.01 | 1,00-1,03 | 0.166 |
| Pre TIMI flow grade | 1.38 | 1.19-1.60 | <0.001 |
| Culprit lesion: LAD | 0.72 | 052-1.00 | 0.050 |
| Onset to balloon time, hour | 0.99 | 0.99-1.00 | 0.033 |
| Door to balloon time, hour | 1.00 | 0.99-1.00 | 0.683 |
| Laboratory Data |  |  |  |
| eGFR | 0.99 | 0.98-1.00 | 0.001 |
| LDL cholesterol, mg/dL | 1.00 | 0.99-1.00 | 0.992 |
| HDL cholesterol, mg/dL | 1.00 | 0.99-1.01 | 0.851 |
| HbA1c, | 1.01 | 0.89-1.15 | 0.833 |
| Hemoglobin, g/dL | 0.98 | 0.91-1.06 | 0.614 |
| BNP, per 10 pg/ml | 1.00 | 0.99-1.01 | 0.219 |
| Prehospital medications |  |  |  |
| ACE-i/ARB | 1.01 | 0.71-1.44 | 0.946 |
| β blocker | 1.15 | 0.72-1.83 | 0.569 |
| MRA | 1.51 | 0.47-4.78 | 0.488 |
| Calcium channel blockers | 1.30 | 0.90-1.88 | 0.156 |
| Diuretics | 0.86 | 0.40-1.87 | 0.702 |
| Statin | 1.53 | 1.07-2.18 | 0.019 |
| Aspirin | 1.06 | 0.73-1.54 | 0.763 |

ACE-i = angiotensin converting enzyme inhibitor ARB = angiotensin receptor blocker; BNP = brain natriuretic peptide; BP-time = time between balloon and peak creatine kinase-myoglobin binding; CI = confidence interval; eGFR = estimated glomerular filtration rate; HbA1c = glycated hemoglobin; LAD = left anterior descending coronary artery; LDL = low-density lipoprotein; LVEF = left ventricle ejection fraction; MRA = mineralocorticoid receptor antagonist; OR = odds ratio; PCI = percutaneous coronary intervention; TIMI = thrombolysis in myocardial infarction trial

**Supplemental Table 5. Multivariate logistic regression model for long BP-time**

|  | **OR** | **95% CI** | ***P* value** |
| --- | --- | --- | --- |
| Age, years | 1.00 | 0.98-1.01 | 0.520 |
| Male | 1.83 | 1.02-3.30 | 0.044 |
| History of dyslipidemia | 1.13 | 0.76-1.69 | 0.530 |
| Previous myocardial infarction | 1.13 | 0.48-2.64 | 0.780 |
| Prior PCI history | 1.03 | 0.50-2.11 | 0.940 |
| Baseline systolic blood pressure, mmHg | 1.00 | 0.99-1.00 | 0.220 |
| Killip classification | 1.30 | 1.04-1.62 | 0.021 |
| Pre TIMI flow grade | 1.39 | 1.17-1.65 | <0.001 |
| LAD culprit lesion | 0.65 | 0.45-0.95 | 0.024 |
| eGFR, mL/min/1.73m^2^ | 1.00 | 0.99-1.01 | 0.64 |
| Door to balloon time, hour | 0.95 | 0.80-1.13 | 0.590 |
| Onset to balloon time, hour | 0.97 | 0.93-1.00 | 0.054 |
| History of administration of statin | 1.40 | 0.91-2.15 | 0.130 |

BP-time = time between balloon and peak creatine kinase-myoglobin binding; CI = confidence interval; eGFR = estimated glomerular filtration rate; LAD = left anterior descending artery; OR = odds ratio; TIMI = thrombolysis in myocardial infarction trial
